# Fibrinolysis: A Primordial System Linked to the Immune Response

**DOI:** 10.3390/ijms22073406

**Published:** 2021-03-26

**Authors:** Robert L. Medcalf, Charithani B. Keragala

**Affiliations:** Molecular Neurotrauma and Haemostasis Laboratory, Australian Centre for Blood Diseases, Central Clinical School Melbourne, Monash University, Melbourne, VIC 3004, Australia; Charithani.Keragala@monash.edu

**Keywords:** fibrinolysis, plasminogen activation, immune response, inflammation

## Abstract

The fibrinolytic system provides an essential means to remove fibrin deposits and blood clots. The actual protease responsible for this is plasmin, formed from its precursor, plasminogen. Fibrin is heralded as it most renowned substrate but for many years plasmin has been known to cleave many other substrates, and to also activate other proteolytic systems. Recent clinical studies have shown that the promotion of plasmin can lead to an immunosuppressed phenotype, in part via its ability to modulate cytokine expression. Almost all immune cells harbor at least one of a dozen plasminogen receptors that allows plasmin formation on the cell surface that in turn modulates immune cell behavior. Similarly, a multitude of pathogens can also express their own plasminogen activators, or contain surface proteins that provide binding sites host plasminogen. Plasmin formed under these circumstances also empowers these pathogens to modulate host immune defense mechanisms. Phylogenetic studies have revealed that the plasminogen activating system predates the appearance of fibrin, indicating that plasmin did not evolve as a fibrinolytic protease but perhaps has its roots as an immune modifying protease. While its fibrin removing capacity became apparent in lower vertebrates these primitive under-appreciated immune modifying functions still remain and are now becoming more recognised.

## 1. Introduction

The plasminogen activating (“fibrinolytic”) system is one of the most important proteolytic cascades in all mammals and indeed in a variety of other species. While conventionally associated with blood clot removal via the generation of the key protease, plasmin, this system also performs a multitude of other important functions, some of which are beginning to impact on clinical medicine. Some of these developments, notably on the actions of plasmin on the immune response have been recently reviewed [1]. Direct evidence is now emerging, not only from animal models, but also in humans that the modulation of the plasminogen activating system does indeed impact on immune function. This review will discuss the link between the plasminogen activating system with immune function and also argue the case that the evolution of this system may have been initially directed at immune modulation but which subsequently became adapted for other functions, including fibrin removal.

## 2. Plasminogen Activation: A Universal System with a Broad Repertoire

For centuries, blood has been known to clot when outside the body. It was also observed that clotted blood could also spontaneously dissolve and this was initially put down to being a result of putrefaction (i.e., just simple protein decay, see [2]). However, in 1893 biochemical studies on fibrin in clotted human blood revealed that the longer blood rested on the bench, the lower the concentration of fibrin that was found [3]. Hence, fibrin was not simply rotting away, but was being removed via some apparent enzymatic process. This finding led to the first coining of the term “fibrinolysis” [3]. The biochemical underpinnings of this newly discovered process were of clear academic interest, but identifying the protease responsible for attacking the fibrin in the blood was not easy. In fact, it took another 40 years before scientists stumbled upon an exogenous source of fibrinolytic activity: the first in the saliva of blood feeding vampire bats by Otto Bier in 1932 [4] and in the same year, Aoi described a “Fibrolytic” activity in isolates of staphylococcus [5] that was subsequently identified as staphylokinase (see [6]). In 1933, a fibrinolytic activity was found in the culture broth of β-haemolytic streptococci [7]. These bacteria were also responsible for severe bleeding complications in patients and this newly identified fibrinolytic activity was the likely culprit. The nuts and bolts of the fibrinolytic pathway were largely revealed using these bacterial sources, mostly from streptococci. Initially the entity found in streptococci was referred to as “fibrinolysin” but was eventually designated as “streptokinase”. Critically important experiments revealed streptokinase (and also staphylokinase) first needed to activate a plasma precursor as first reported by Milstone in 1941 [8]. This plasma “zymogen” was referred to as “pro-fibrinolysin” (later renamed as plasminogen) and the active fibrinolytic form as “fibrinolysin” (i.e., plasmin); see [9]. In the meantime, the elusive human-derived plasminogen activators were subsequently described, the first in 1947 [10] that was detected in human cells (initially referred to as tissue-cytofibrinolysin, and later as tissue-type plasminogen activator; t-PA) while a urinary source (urokinase-type plasminogen activator; u-PA) was described in 1952 [11]. While the fundamental studies using streptokinase and staphylokinase were ground breaking, mechanistically these bacterial entities were later found to function very differently to the renowned human plasminogen activators, t-PA and uPA. Indeed, neither streptokinase nor staphylokinase are plasminogen activators nor kinases. In fact, they are not even proteases but are rather plasminogen binding proteins: when complexed with plasminogen, the resulting complex itself becomes a plasminogen activator [12].

During this important period in fibrinolysis research, it did not take long to consider the possibility that harnessing this process might be of clinical use in patients with thromboembolic disease. Indeed, streptokinase was first used clinically in 1949 (in patients with pleural exudates [13]) and continues to be used today being listed as an essential medicine by the World Health Organisation (WHO). Staphylokinase was also pursued and in later years was shown to have distinct benefits over streptokinase; see [14]. Both tPA and uPA are also widely used agents and therapeutic thrombolysis is now mainstream in clinical medicine.

On the other side of the coin, it had become apparent early on that excessive fibrinolysis could promote the premature removal of blood clots and cause devastating bleeding. This led to the development of anti-fibrinolytic agents [15], notably tranexamic acid (TXA) in the early 1960′s, that is also listed today as an essential medicine by the WHO. Therefore, after the serendipitous finding that a fibrinolytic process existed in 1893 and the subsequent laboratory studies that followed into the mid 1940′s, mainstream medicine eventually exploited this enzymatic pathway for clinical benefit either to remove clots by forcibly generating plasmin, or by blocking plasmin generation or activity to preserve blood clots and stop bleeding, even to this day.

It would seem that this story could simply end here, and it probably could if not for the fact that the plasminogen activating process did not evolve for the sole purpose of removing fibrin. It also seems an anomaly that the renowned endogenous human plasminogen activators (t-PA and u-PA) are relatively specific for plasminogen, yet the formed plasmin is not at all solely specific for fibrin. Indeed, the promiscuous, trypsin-like substrate specificity of plasmin has empowered it with the capacity to cleave many targets. Hence, the target specificity of plasmin is derived from the plasminogen activators themselves that decide on where plasmin is produced. However, depending on the location and circumstances, plasmin has the potential to act upon numerous targets and in doing so influence a variety of physiological and even pathological processes.

Adding more complexity to this is that the list of plasminogen activators has expanded quite impressively. Even in humans, plasminogen can be activated by other serine proteases including kallikrein [16] and complement [17] while thrombin can have bidirectional effects on plasminogen activation [18]. However, there is an even longer list of proteases that activate human plasminogen from various pathogens as discussed below in Section 4.

Plasminogen itself is expressed at high concentration in human plasma (2 μM), but it is also present in essentially every other compartment at varying levels, ranging from seminal fluid [19] to the central nervous system [20] (and see [21]). The primary inhibitor of plasmin, α2-antiplasmin, is also expressed in many of these locations [22,23]. Nonetheless, with the range of plasminogen activators available from human and non-human sources (below), plasmin has the potential to be produced and to be largely controlled by antiplasmin (and by other molecules) almost anywhere, including locations where fibrin is not even present.

It is not surprising that the broad spectrum of plasmin substrates has implicated the plasminogen activating system in many processes ranging from wound repair to synaptic plasticity [24]. There is extensive literature that has summarized these non-canonical activities in the early 2000′s [25,26] and in more recent times [1,17,27,28,29]. However, one key function that may underpin the broad role of this system is its effect on the immune response. When reflecting on the early findings of the pioneers in this field, a link between fibrinolysis and the immune response was evident at the very outset but its profound effect at removing fibrin and the subsequent clinical development of fibrinolytic and anti-fibrinolytic agents overshadowed any other possible role. Additionally, off-target effects of fibrinolytic agents in clinical medicine were essentially only related to bleeding (which was predicted), while side-effects of the use of anti-fibrinolytic agents were generally negligible [30,31]. However, then again, any possible effects on immune function were not on the radar anyway. Additionally, off-target effects may not necessarily be deleterious anyway and would go undetected.

## 3. Plasminogen, Fibrinolysis and Immune Function

Plasmin(ogen) is now appreciated for its role as an immune modulator interacting directly with numerous cells of the immune response including monocytes, macrophages and dendritic cells [1]. Plasmin, formed on the surface of immune cells, is capable of activating several pro-inflammatory pathways resulting in cytokine production [32]. Even in healthy humans, simply administering an antifibrinolytic agent results in a significant increase in pro-inflammatory cytokines within 2h of treatment [33] suggesting that inherent plasmin formation provides a means to keep inflammation repressed, but this can quickly change as soon as plasmin is inhibited. This further implicates the entire plasminogen activation system as an essential part of an innate surveillance network geared to respond to immune challenges. Much of this immune/inflammatory signalling is mediated by at least 12 plasminogen receptors that are differentially expressed on the cell surface of almost all immune cells [34]. For example, cell surface-bound plasmin(ogen) is required for the efficient migration of macrophages to sites of inflammation [35]. Plasminogen has also been shown to promote macrophage phagocytosis in mice, with plasminogen-deficient mice exhibiting a 60% delay in clearing apoptotic thymocytes by spleen and an 85% reduction in uptake of immunoglobulin opsonised bodies by peritoneal macrophages [36]. Phagocytosis of antibody-mediated erythrocyte clearance by liver Kupffer cells was also reduced in plasminogen-deficient mice compared to Plg+/+ mice. Gene array studies of tissues from these Plg−/− mice revealed downregulation of several genes involved in phagocytosis, suggesting that plasminogen may be able to change the expression of certain genes contributing to phagocytosis [36]. Plasmin also engages other elements of innate immunity including the complement cascade, components of the extracellular matrix as wells as cells of the vasculature including endothelial and smooth muscle cells [1]. In stark contrast to its pro-inflammatory properties, plasmin also exhibits several anti-inflammatory and immunosuppressive responses. Plasmin(ogen) appears to regulate, via its receptor, annexin A1, key aspects of inflammation resolution, in particular, macrophage reprogramming, neutrophil apoptosis and efferocytosis [37]. Plasmin-treated dendritic cells for instance fail to undergo maturation following phagocytosis, exhibit reduced migration to lymph nodes and also stimulate the release of significant amounts of transforming growth factor-β (TGF-β) which has immunosuppressive properties. These dendritic cells also have reduced ability to induce allogeneic lymphocyte proliferation. These properties of plasmin are important in maintaining tissue homeostasis where it aids in initiating a response to tissue injury while preventing self-reactivity/autoimmunity [36,38].

Hence, it is apparent that pathogens, by harnessing the plasminogen activating pathway, might gain an additional advantage by counteracting immune defense processes that are initiated, at least in part, by cell-surface plasmin generation. While there is clearly an argument that these pathogens would survive host defenses by clearing fibrin, there is now looming evidence that also suggests that the hijacking of host plasminogen might aid pathogen survival by using plasmin to disengage some of the key immune pathways as an effective countermeasure.

## 4. Microorganisms, Plasmin Formation and Fibrinolysis

As mentioned above, streptokinase and staphylokinase were discovered as a key molecule released from some strains of β-haemolytic streptococci and staphylococcus, respectively. However, this is not unique to these particular strains as similar molecules are produced by many other bacteria (below). Even if a molecule is not produced endogenously, bacteria almost universally have the capacity to bind plasminogen and to use the host plasminogen activators as a means to generate localized plasmin. The formed plasmin is harnessed by these pathogens not only to remove the confines of fibrin, but also to suppress the host immune response and evade local immune attack [39]. Over 40 binding proteins have been reported in bacterial species which target plasmin(ogen) [40]. For example, Mycobacterium tuberculosis has 13 proteins with plasminogen binding potential and *Mycoplasma pneumoniae* exhibits 6 [41,42].

Plasminogen binding to these proteins activates a variety of mechanisms aimed at infiltrating host defences. These include for example the degradation of extracellular matrix proteins by *Leptospira*, where urokinase (u-PA) activates bound plasminogen to plasmin which then degrades fibronectin and laminin [43]. Remarkably, streptokinase of the non-human streptococci show evolutionary species-specificity for the plasminogen of the animal host they infect [44,45,46]. Staphylokinase, secreted by *Staphyloccocus aureus*, exhibits high affinity binding to plasminogen in plasma, thus forming a complex that can effectively activate plasminogen (akin to streptokinase) while also evading the inactivating capacity of α2-antiplasmin by binding to fibrin [14]. Interestingly, *Yersinia Pestis*, the causative pathogen of the plague, which killed a third of the European population in the 14th century, expresses a plasmid gene *pla* which encodes a surface plasminogen activator protease with unusual kinetic properties. The expression of this protease increases the virulence of *Yersinia Pestis* and is also likely to cleave and inactivate plasminogen activator inhibitor-1 (PAI-1), increasing the conversion of plasminogen to plasmin and promote virulence in the host [47,48,49].

Plasminogen-dependent extracellular matrix degeneration is utilised by the main pathogens causing bacterial meningitis, *H. influenzae, S. pneumonia* and *N. meningitides* [44,50]. Furthermore, plasmin’s proteolytic role is harnessed by bacteria in degrading plasma proteins and peptides, including complement and immunoglobulins, which are critical in antigen presentation and processing within the host innate immune repertoire [51]. For example, *Leptospira* surface protein Lsa23 not only has the ability to block activation of both the alternative and classical complement pathways, but binds to and activates plasminogen to plasmin which in turn degrades complement proteins C3b and C4b, together improving the chances of evading host immunity [52].

Plasminogen receptors are also expressed on fungi including several Candida species, *Aspergillus, Cryptococcus neoformans* and *Pneumocystis carinii* [53,54]. Many of the receptors on Cryptococcus have the ability to activate the host PA system to allow the fungus to cross tissue barriers including the critical blood–brain barrier [55].

Plasminogen is also important in the invasiveness and pathogenesis of several parasites. *Trypanosoma cruzi, Leishmania* and the malarial parasites *Plasmodium falciparum* are known to engage enolase-plasminogen binding as well as uPA in aspects of their pathogenicity and replication [56,57]. More recently, the fibrinolytic system was reported to be essential for parasite migration across the dermis and liver [58]. Helminth parasites also exhibit multiple plasminogen binding proteins as they are in contact with fibrinolytic proteins within the intravascular space. Recruitment of plasminogen on the worm’s surface appears to be one method of host immune evasion [40].

Most of the discussion above relates to the consequences of plasmin in modulating immune surveillance and how this can be intercepted by pathogens. There is also evidence that the plasminogen activators themselves, and independently of activating plasminogen, can also modulate immune function. Indeed, catalytically inactive t-PA has been reported to express inflammatory mediators by macrophages in vitro in a process dependent on LRP-1 [59]. Another report from the same group implicated a key role for NMDA-1 receptor signalling in this process and also reported that inactive tPA could block LPS toxicity in vivo in mice [60]. This same group just recently indicated that enzymatically inactive t-PA was also protective in a mouse model of inflammatory bowel disease [61].

A summary of the variety of effects of the fibrinolytic system on the immune and inflammatory responses is presented in Table 1.

## 5. Phylogenetic Links with Plasminogen Activation

Phylogenetic studies have further provided compelling evidence to suggest that plasminogen and the plasminogen activators did not co-evolve but eventually became brothers in arms perhaps as a matter of convenience. Although plasmin, t-PA and u-PA are serine proteases, the codon usage used for the active serine in plasminogen (AGT) differs at two position to that used to encode the serine in t-PA (TCG) and u-PA (TCA) [81]. This strongly suggests that within the serine protease family, plasminogen evolved separately (and earlier, see below) from u-PA and t-PA

It has also been reported that the primordial ancestor of plasminogen first appeared in protochordates (e.g., amphioxus, sea squirt and related species) [82,83], animals that contain hemolymph that does not even clot but which cross-reacts with anti-human plasminogen antibodies and was localized to the hepatic diverticulum [84]. Plasminogen cDNA was cloned from Amphioxus *B. belcheri* and expressed in *E. coli*. Studies on this recombinant protein indicated that it contained two kringle domains in the N-terminus (not five as in humans) and a serine protease domain in the N-terminus. This molecule also lacked the PAN domain [85]. It also appeared that a lysine binding domain was conserved in one of these kringles [85]. Moreover, the amphioxus plasminogen harboured the putative t-PA/u-PA cleavage site (Arg-Val). The catalytic triad (His-Asp-Ser), critical for protease function was also present and located at positions corresponding to human plasminogen. Consistent with these finding the amphioxus plasminogen was shown to generate plasmin when incubated with human uPA [85]. It is not clear what endogenous proteases were used to activate plasminogen, since the classical plasminogen activators, t-PA and u-PA, only appeared around 20 million years later in cartilaginous fish, together with PAI-1 (see [83]). While protochordates cannot generate fibrin, they do contain a primitive yet full length fibrinogen molecule that does not harbour thrombin cleavage sites [86]. Hence, the primary function of this early plasminogen/plasmin molecule had nothing to do with fibrinolysis per se, yet it remains possible that the co-existing primitive fibrinogen itself could have been a substrate for this early plasmin. As cell clumping had been observed in sea squirts, it was speculated by Russell Doolittle that this fibrinogen molecule may have been involved in mediating cell–cell interactions and perhaps having some innate immune function [86]. It is possible that plasminogen had some regulatory function with fibrinogen that also may have had some relationship with ancestral complement proteins that were also present in these animals.

As biological complexity evolved with the appearance of vertebrates that included a sophisticated clotting system, existing molecules gained new or additional enzymatic functions (perhaps even re-purposed) to generate and to keep fibrin and other proteins in check. It is acknowledged that caution is needed in extrapolating primitive functions and the evolutionary time course of fibrin-targeted proteases using extant species. Nonetheless, the finding that a bonafide plasminogen exists in an extant species that does not make fibrin is indicative of another in vivo role of plasminogen, which may have been a primitive immune function.

## 6. Conclusions, Clinical Implications and Future Potential

There is no doubt about the importance of the fibrinolytic system in the removal of fibrin. Indeed, modulation of this process, either by increasing or decreasing plasmin levels has critically important effects in clinical medicine in relation to the removal of occlusive clots, or to reduce bleeding, respectively. It could be well argued that fibrin is the most relevant substrate for plasmin in the setting of thrombosis simply due to the mass of fibrin that accumulates and the risk that this poses. Under physiological conditions, where fibrin formation is at background levels, plasmin formation still occurs. Plasmin is a promiscuous protease, so while it might still be performing some degree of fibrinolysis under normal conditions, its broad substrate specificity empowers it to cleave other targets, some of which act in an immune surveillance capacity modulating inflammation. This is apparent from studies on volunteers administered antifibrinolytic drugs where baseline levels of proinflammatory cytokines rapidly increase [33]. There are now many other reports implicating plasmin, tPA and in fact the entire fibrinolytic system in the immune response. These immune/inflammatory functions may indeed have been the original role of this enzymatic system given that plasmin generation occurs in lower order species (protochordates) where fibrin itself is absent. Understanding the evolutionary origins of the plasminogen activating system and the realization that perhaps it is not as fibrin centric as initially thought reveals opportunities to apply and harness these properties for means not previously considered. Could thrombolysis in acute ischaemic stroke impair the host immune response in a plasmin-mediated manner while also running the risk of intracerebral haemorrhage? Could antifibrinolytic therapy in major surgery confer an immune advantage in recovering patients while also avoiding excessive bleeding? The answers to these questions are slowly emerging as is the potential for the wider implications of plasmin(ogen) activation beyond just haemostasis, influencing scientific research, clinical practice and ultimately patient outcomes. Further research aimed at harnessing the plasminogen activating system for its immune modulatory properties may potentially open doors to understanding this unique process further.

## Figures and Tables

**Table 1:** Properties of plasmin(ogen) in inflammation and immunity.

Target Effects	Properties of Plasmin(ogen)	References
***Proinflammatory*** **Macrophage and Monocyte effects**	Interacts with macrophage migration and activation via plasminogen receptors. Promotes cytokine production in macrophages.	[32,35,62]
	Directly alters gene expression in macrophages by binding plasminogen receptors and enhancing phagocytic capacity, efferocytosis and foam cell formation.	[37,63,64]
	Promote macrophage phagocytosis in mice	[36]
	Potent chemoattractant of monocytes, induces actin polymerisation.	[65]
	Activates 5-lipoxygenase pathway in monocytes and macrophages resulting in the synthesis of proinflammatory leukotrienes.	[66]
	Stimulates JAK/STAT signalling in monocytes resulting in MCP-1 release, further promoting monocyte recruitment.	[67]
	Increases phagocytic activity of DCs without causing activation.	[38]
**Dendritic Cell (DC)** **effects**	This interaction is involved in the chemoattraction of dendritic cells, T- and B cells. Triggers chemotaxis of monocyte derived DCs and a T helper type-1 (Th1) phenotype in CD4+ T cells.	[68]
	Indirectly promotes neutrophil recruitment by binding to mast cells and stimulating release of leukotrienes	[69]
	Induce expression of CCR6-activating chemokine CCL20 in dermis via induction of NF-kB.	[70]
**Other inflammatory** **effects**	Stimulates NF-kB and AP-1, resulting in the production of tumor necrosis factor (TNF)-α, interleukin (IL)-1α, IL-1β, and tissue factor.	[71]
	Activates phospholipase A2 in endothelial cells, releasing arachidonic acid and subsequent production of prostacyclin, enhanced nitric oxide (NO)-mediated vasorelaxation and chemotactic monocyte chemotactic protein (MCP)-1 release.	[39,72,73]
	In pulmonary epithelial cells, plasmin induces cyclooxygenase (COX)-2, resulting in the release of antifibrotic prostaglandin E-2 (PGE-2).	[74]
	Promotes complement activation.	[17,75]
	Binds to platelets via PAR-4 and dose-dependently activate or inhibit platelet activation and aggregation. Interacts with extracellular matrix, endothelial cells, smooth muscle.	[76,77]
	Plasmin can activate the Matrix Metalloproteinases, transforming growth factor (TGF)-β, and neurotrophic factors.	[78,79,80]
	Inhibition of DC maturation following phagocytosis thereby inducing a tolerogenic phenotype. Reduced migration of DCs to lymph nodes and increase release of TGF-β. Reduction in DC ability to induce allogeneic lymphocyte proliferation.	[38]
***Immuno-suppression*** **DC effects**	Suppression of proinflammatory cytokines in vivo, reversed by tranexamic acid. Inhibition of plasmin reduces post-surgical infection rates.	[33]

## References

[1] Draxler D.F., Sashindranath M., Medcalf R.L. (2017). Plasmin: A Modulator of Immune Function. Semin. Thromb. Hemost..

[2] Green J.R. (1887). Note on the Action of Sodium Chloride in dissolving Fibrin. J. Physiol..

[3] Dastre A. (1893). Fibrinolyse dans le sang. Arch. Physiol..

[4] Bier O.E. (1932). Action anticoagulante et fibrinolytique de l’extract des glands salivaires d’une chauve-souris hematophage (*desmodus rufus*). C.R. Soc Biol. (Paris).

[5] Aoi F. (1932). On the fibrolysis of the staphylococcus. Kitasato Arch. Exptl. Med..

[6] Lack C.H. (1948). Staphylokinase: An activator of plasma Protease. Nature.

[7] Tillett W.S., Garner R.L. (1933). The Fibrinolytic Activity of Hemolytic Streptococci. J. Exp. Med..

[8] Milstone H. (1941). A factor in normal human blood which particiaptes in streptococcal fibrinolysis. J. Immunol..

[9] Macfarlane R.G., Pilling J. (1946). Observations on fibrinolysis; plasminogen, plasmin, and antiplasmin content of human blood. Lancet.

[10] Astrup T., Permin P.M. (1947). Fibrinolysis in the animal organism. Nature.

[11] Sobel G.W., Mohler S.R., Jones N.W., Dowdy A.B.C., Guest M.M. (1952). Urokinase: An activator of plasma profibrinolysin extracted from urine. Am. J. Physiol..

[12] Parry M.A., Zhang X.C., Bode I. (2000). Molecular mechanisms of plasminogen activation: Bacterial cofactors provide clues. Trends Biochem. Sci..

[13] Tillett W.S., Sherry S. (1949). The effect in patients of streptococcal fibrinolysin and streptococcal desoxyribonuclease on fibrinous, purulent, and sanguinous pleural exudations. J. Clin. Investig..

[14] Lijnen H.R., Van Hoef B., De Cock F., Okada K., Ueshima S., Matsuo O., Collen D. (1991). On the mechanism of fibrin-specific plasminogen activation by staphylokinase. J. Biol. Chem..

[15] Okamoto S.O.U. (1962). A new potent antifibrinolytic substance and its effects on blood of animals. Keio J. Med..

[16] Jorg M., Binder B.R. (1985). Kinetic analysis of plasminogen activation by purified plasma kallikrein. Thromb. Res..

[17] Foley J.H. (2017). Plasmin(ogen) at the Nexus of Fibrinolysis, Inflammation, and Complement. Semin. Thromb. Hemost..

[18] Tomczyk M., Suzuki Y., Sano H., Brzoska T., Tanaka H., Urano T. (2016). Bidirectional functions of thrombin on fibrinolysis: Evidence of thrombin-dependent enhancement of fibrinolysis provided by spontaneous plasma clot lysis. Thromb. Res..

[19] Lwaleed B.A., Greenfield R., Stewart A., Birch B., Cooper A.J. (2004). Seminal clotting and fibrinolytic balance: A possible physiological role in the male reproductive system. Thromb. Haemost..

[20] Basham M.E., Seeds N.W. (2001). Plasminogen expression in the neonatal and adult mouse brain. J. Neurochem..

[21] Zhang L., Seiffert D., Fowler B.J., Jenkins G.R., Thinnes T.C., Loskutoff D.J., Parmer R.J., Miles L.A. (2002). Plasminogen Has a Broad Extrahepatic Distribution. Thromb. Haemost..

[22] Del Giudice P.T., da Silva B.F., Lo Turco E.G., Fraietta R., Spaine D.M., Santos L.F., Pilau E.J., Gozzo F.C., Cedenho A.P., Bertolla R.P. (2013). Changes in the seminal plasma proteome of adolescents before and after varicocelectomy. Fertil. Steril..

[23] Kawashita E., Kanno Y., Asayama H., Okada K., Ueshima S., Matsuo O., Matsuno H. (2013). Involvement of alpha2-antiplasmin in dendritic growth of hippocampal neurons. J. Neurochem..

[24] Medcalf R.L. (2017). Fibrinolysis: From blood to the brain. J. Thromb. Haemost..

[25] Yepes M., Lawrence D.A. (2004). New functions for an old enzyme: Nonhemostatic roles for tissue-type plasminogen activator in the central nervous system. Exp. Biol. Med. (Maywood).

[26] Samson A.L., Medcalf R.L. (2006). Tissue-type plasminogen activator: A multifaceted modulator of neurotransmission and synaptic plasticity. Neuron.

[27] Draxler D.F., Medcalf R.L. (2015). The fibrinolytic system-more than fibrinolysis?. Transfus. Med. Rev..

[28] Fredriksson L., Lawrence D.A., Medcalf R.L. (2017). tPA Modulation of the Blood-Brain Barrier: A Unifying Explanation for the Pleiotropic Effects of tPA in the CNS. Semin. Thromb. Hemost..

[29] Medcalf R.L., Keragala C., Draxler D.F. (2019). Fibrinolysis and the immune response in trauma. Semin. Thromb. Haemost..

[30] Warnaar N., Mallett S.V., Klinck J.R., de Boer M.T., Rolando N., Burroughs A.K., Jamieson N.V., Rolles K., Porte R.J. (2009). Aprotinin and the risk of thrombotic complications after liver transplantation: A retrospective analysis of 1492 patients. Liver Transpl..

[31] Henry D.A., Carless P.A., Moxey A.J., O’Connell D., Stokes B.J., Fergusson D.A., Ker K. (2011). Anti-fibrinolytic use for minimising perioperative allogeneic blood transfusion. Cochrane Database Syst. Rev..

[32] Li Q., Laumonnier Y., Syrovets T., Simmet T. (2007). Plasmin triggers cytokine induction in human monocyte-derived macrophages. Arter. Thromb Vasc Biol.

[33] Draxler D.F., Yep K., Hanafi G., Winton A., Daglas M., Ho H., Sashindranath M., Wutzlhofer L.M., Forbes A., Goncalves I. (2019). Tranexamic acid modulates the immune response and reduces postsurgical infection rates. Blood Adv..

[34] Miles L.A., Parmer R.J. (2013). Plasminogen receptors: The first quarter century. Semin Thromb Hemost.

[35] Silva L.M., Lum A.G., Tran C., Shaw M.W., Gao Z., Flick M.J., Moutsopoulos N.M., Bugge T.H., Mullins E.S. (2019). Plasmin-mediated fibrinolysis enables macrophage migration in a murine model of inflammation. Blood.

[36] Das R., Ganapathy S., Settle M., Plow E.F. (2014). Plasminogen promotes macrophage phagocytosis in mice. Blood.

[37] Sugimoto M.A., Ribeiro A.L.C., Costa B.R.C., Vago J.P., Lima K.M., Carneiro F.S., Ortiz M.M.O., Lima G.L.N., Carmo A.A.F., Rocha R.M. (2017). Plasmin and plasminogen induce macrophage reprogramming and regulate key steps of inflammation resolution via annexin A1. Blood.

[38] Borg R.J., Samson A.L., Au A.E., Scholzen A., Fuchsberger M., Kong Y.Y., Freeman R., Mifsud N.A., Plebanski M., Medcalf R.L. (2015). Dendritic Cell-Mediated Phagocytosis but Not Immune Activation Is Enhanced by Plasmin. PLoS ONE.

[39] Syrovets T., Lunov O., Simmet T. (2012). Plasmin as a proinflammatory cell activator. J. Leukoc. Biol..

[40] Ayon-Nunez D.A., Fragoso G., Bobes R.J., Laclette J.P. (2018). Plasminogen-binding proteins as an evasion mechanism of the host’s innate immunity in infectious diseases. Biosci. Rep..

[41] Grundel A., Friedrich K., Pfeiffer M., Jacobs E., Dumke R. (2015). Subunits of the Pyruvate Dehydrogenase Cluster of Mycoplasma pneumoniae Are Surface-Displayed Proteins that Bind and Activate Human Plasminogen. PLoS ONE.

[42] Xolalpa W., Vallecillo A.J., Lara M., Mendoza-Hernandez G., Comini M., Spallek R., Singh M., Espitia C. (2007). Identification of novel bacterial plasminogen-binding proteins in the human pathogen Mycobacterium tuberculosis. Proteomics.

[43] Vieira M.L., Atzingen M.V., Oliveira R., Mendes R.S., Domingos R.F., Vasconcellos S.A., Nascimento A.L. (2012). Plasminogen binding proteins and plasmin generation on the surface of Leptospira spp.: The contribution to the bacteria-host interactions. J. Biomed. Biotechnol..

[44] Peetermans M., Vanassche T., Liesenborghs L., Lijnen R.H., Verhamme P. (2016). Bacterial pathogens activate plasminogen to breach tissue barriers and escape from innate immunity. Crit. Rev. Microbiol..

[45] Sun H., Ringdahl U., Homeister J.W., Fay W.P., Engleberg N.C., Yang A.Y., Rozek L.S., Wang X., Sjobring U., Ginsburg D. (2004). Plasminogen is a critical host pathogenicity factor for group A streptococcal infection. Science.

[46] Gladysheva I.P., Turner R.B., Sazonova I.Y., Liu L., Reed G.L. (2003). Coevolutionary patterns in plasminogen activation. Proc. Natl. Acad. Sci. USA.

[47] Eddy J.L., Schroeder J.A., Zimbler D.L., Caulfield A.J., Lathem W.W. (2016). Proteolysis of plasminogen activator inhibitor-1 by Yersinia pestis remodulates the host environment to promote virulence. J. Thromb. Haemost..

[48] Sodeinde O.A., Subrahmanyam Y.V., Stark K., Quan T., Bao Y., Goguen J.D. (1992). A surface protease and the invasive character of plague. Science.

[49] Banerjee S.K., Crane S.D., Pechous R.D. (2020). A Dual Role for the Plasminogen Activator Protease During the Preinflammatory Phase of Primary Pneumonic Plague. J. Infect. Dis..

[50] Knaust A., Weber M.V., Hammerschmidt S., Bergmann S., Frosch M., Kurzai O. (2007). Cytosolic proteins contribute to surface plasminogen recruitment of Neisseria meningitidis. J. Bacteriol..

[51] Crane D.D., Warner S.L., Bosio C.M. (2009). A novel role for plasmin-mediated degradation of opsonizing antibody in the evasion of host immunity by virulent, but not attenuated, Francisella tularensis. J. Immunol..

[52] Siqueira G.H., Atzingen M.V., de Souza G.O., Vasconcellos S.A., Nascimento A. (2016). Leptospira interrogans Lsa23 protein recruits plasminogen, factor H and C4BP from normal human serum and mediates C3b and C4b degradation. Microbiology (Reading).

[53] Crowe J.D., Sievwright I.K., Auld G.C., Moore N.R., Gow N.A., Booth N.A. (2003). Candida albicans binds human plasminogen: Identification of eight plasminogen-binding proteins. Mol. Microbiol..

[54] Funk J., Schaarschmidt B., Slesiona S., Hallstrom T., Horn U., Brock M. (2016). The glycolytic enzyme enolase represents a plasminogen-binding protein on the surface of a wide variety of medically important fungal species. Int. J. Med. Microbiol..

[55] Stie J., Fox D. (2012). Blood-brain barrier invasion by Cryptococcus neoformans is enhanced by functional interactions with plasmin. Microbiology (Reading).

[56] Vanegas G., Quinones W., Carrasco-Lopez C., Concepcion J.L., Albericio F., Avilan L. (2007). Enolase as a plasminogen binding protein in Leishmania mexicana. Parasitol. Res..

[57] Roggwiller E., Fricaud A.C., Blisnick T., Braun-Breton C. (1997). Host urokinase-type plasminogen activator participates in the release of malaria merozoites from infected erythrocytes. Mol. Biochem. Parasitol..

[58] Alves E.S.T.L., Radtke A., Balaban A., Pascini T.V., Pala Z.R., Roth A., Alvarenga P.H., Jeong Y.J., Olivas J., Ghosh A.K. (2021). The fibrinolytic system enables the onset of Plasmodium infection in the mosquito vector and the mammalian host. Sci. Adv..

[59] Mantuano E., Brifault C., Lam M.S., Azmoon P., Gilder A.S., Gonias S.L. (2016). LDL receptor-related protein-1 regulates NFkappaB and microRNA-155 in macrophages to control the inflammatory response. Proc. Natl. Acad. Sci. USA.

[60] Mantuano E., Azmoon P., Brifault C., Banki M.A., Gilder A.S., Campana W.M., Gonias S.L. (2017). Tissue-type plasminogen activator regulates macrophage activation and innate immunity. Blood.

[61] Das L., Banki M.A., Azmoon P., Pizzo D., Gonias S.L. (2021). Enzymatically Inactive Tissue-Type Plasminogen Activator Reverses Disease Progression in the Dextran Sulfate Sodium Mouse Model of Inflammatory Bowel Disease. Am. J. Pathol..

[62] Thaler B., Baik N., Hohensinner P.J., Baumgartner J., Panzenbock A., Stojkovic S., Demyanets S., Huk I., Rega-Kaun G., Kaun C. (2019). Differential expression of Plg-RKT and its effects on migration of proinflammatory monocyte and macrophage subsets. Blood.

[63] Das R., Ganapathy S., Mahabeleshwar G.H., Drumm C., Febbraio M., Jain M.K., Plow E.F. (2013). Macrophage gene expression and foam cell formation are regulated by plasminogen. Circulation.

[64] Vago J.P., Sugimoto M.A., Lima K.M., Negreiros-Lima G.L., Baik N., Teixeira M.M., Perretti M., Parmer R.J., Miles L.A., Sousa L.P. (2019). Plasminogen and the Plasminogen Receptor, Plg-RKT, Regulate Macrophage Phenotypic, and Functional Changes. Front. Immunol..

[65] Syrovets T., Tippler B., Rieks M., Simmet T. (1997). Plasmin is a potent and specific chemoattractant for human peripheral monocytes acting via a cyclic guanosine monophosphate-dependent pathway. Blood.

[66] Weide I., Tippler B., Syrovets T., Simmet T. (1996). Plasmin is a specific stimulus of the 5-lipoxygenase pathway of human peripheral monocytes. Thromb. Haemost..

[67] Burysek L., Syrovets T., Simmet T. (2002). The serine protease plasmin triggers expression of MCP-1 and CD40 in human primary monocytes via activation of p38 MAPK and janus kinase (JAK)/STAT signaling pathways. J. Biol. Chem..

[68] Li X., Syrovets T., Genze F., Pitterle K., Oberhuber A., Orend K.H., Simmet T. (2010). Plasmin triggers chemotaxis of monocyte-derived dendritic cells through an Akt2-dependent pathway and promotes a T-helper type-1 response. Arterioscler. Thromb. Vasc. Biol..

[69] Uhl B., Zuchtriegel G., Puhr-Westerheide D., Praetner M., Rehberg M., Fabritius M., Hessenauer M., Holzer M., Khandoga A., Furst R. (2014). Tissue plasminogen activator promotes postischemic neutrophil recruitment via its proteolytic and nonproteolytic properties. Arter. Thromb. Vasc. Biol..

[70] Li Q., Ke F., Zhang W., Shen X., Xu Q., Wang H., Yu X.Z., Leng Q., Wang H. (2011). Plasmin plays an essential role in amplification of psoriasiform skin inflammation in mice. PLoS ONE.

[71] Syrovets T., Jendrach M., Rohwedder A., Schule A., Simmet T. (2001). Plasmin-induced expression of cytokines and tissue factor in human monocytes involves AP-1 and IKKbeta-mediated NF-kappaB activation. Blood.

[72] Chang W.C., Shi G.Y., Chow Y.H., Chang L.C., Hau J.S., Lin M.T., Jen C.J., Wing L.Y., Wu H.L. (1993). Human plasmin induces a receptor-mediated arachidonate release coupled with G proteins in endothelial cells. Am. J. Physiol..

[73] Fujiyoshi T., Hirano K., Hirano M., Nishimura J., Takahashi S., Kanaide H. (2007). Plasmin induces endothelium-dependent nitric oxide-mediated relaxation in the porcine coronary artery. Arterioscler. Thromb. Vasc. Biol..

[74] Bauman K.A., Wettlaufer S.H., Okunishi K., Vannella K.M., Stoolman J.S., Huang S.K., Courey A.J., White E.S., Hogaboam C.M., Simon R.H. (2010). The antifibrotic effects of plasminogen activation occur via prostaglandin E2 synthesis in humans and mice. J. Clin. Invest..

[75] Zhao X.J., Larkin T.M., Lauver M.A., Ahmad S., Ducruet A.F. (2017). Tissue plasminogen activator mediates deleterious complement cascade activation in stroke. PLoS ONE.

[76] Quinton T.M., Kim S., Derian C.K., Jin J., Kunapuli S.P. (2004). Plasmin-mediated activation of platelets occurs by cleavage of protease-activated receptor 4. J. Biol. Chem..

[77] Syrovets T., Simmet T. (2004). Novel aspects and new roles for the serine protease plasmin. Cell Mol. Life Sci..

[78] Lijnen H.R. (2002). Matrix metalloproteinases and cellular fibrinolytic activity. Biochemistry (Mosc.).

[79] Pang P.T., Teng H.K., Zaitsev E., Woo N.T., Sakata K., Zhen S., Teng K.K., Yung W.H., Hempstead B.L., Lu B. (2004). Cleavage of proBDNF by tPA/plasmin is essential for long-term hippocampal plasticity. Science.

[80] Yee J.A., Yan L., Dominguez J.C., Allan E.H., Martin T.J. (1993). Plasminogen-dependent activation of latent transforming growth factor beta (TGF beta) by growing cultures of osteoblast-like cells. J. Cell Physiol..

[81] Brenner S. (1988). The molecular evolution of genes and proteins: A tale of two serines. Nature.

[82] Doolittle R.F. (2009). Step-by-step evolution of vertebrate blood coagulation. Cold Spring Harb. Symp. Quant. Biol..

[83] Chana-Munoz A., Jendroszek A., Sonnichsen M., Wang T., Ploug M., Jensen J.K., Andreasen P.A., Bendixen C., Panitz F. (2019). Origin and diversification of the plasminogen activation system among chordates. BMC Evol. Biol..

[84] Liang Y.J., Zhang S.C. (2006). Demonstration of plasminogen-like protein in amphioxus with implications for the origin of vertebrate liver. Acta Zool..

[85] Liu M., Zhang S. (2009). A kringle-containing protease with plasminogen-like activity in the basal chordate Branchiostoma belcheri. Biosci. Rep..

[86] Doolittle R.F. (2012). The protochordate Ciona intestinalis has a protein like full-length vertebrate fibrinogen. J. Innate Immun..

